# Catecholaminergic polymorphic ventricular tachycardia and early-onset atrial fibrillation in a tactical athlete with a heterozygous truncating variant in *TRDN*

**DOI:** 10.1016/j.hrcr.2025.01.019

**Published:** 2025-02-07

**Authors:** Aashish Katapadi, Thomas M. Roston, Andrew D. Krahn, Naga Venkata K. Pothineni, Dhanunjaya Lakkireddy, Douglas Darden

**Affiliations:** 1 Kansas City Heart Rhythm Institute, Overland Park, Kansas; 2 Division of Cardiology and Centre for Cardiovascular Innovation, The University of British Columbia, Vancouver, British Columbia, Canada

**Keywords:** Catecholaminergic polymorphic ventricular tachycardia, Exercise-induced ventricular tachycardia, Triadin gene, *TRDN* variation, Atrial fibrillation, Channelopathy, Genetic screening

**Key Teaching Points**

• Disease-causing biallelic variants in *TRDN* cause disturbances in calcium handling within cardiac myocytes, predisposing individuals to severe arrhythmias.
• Managing catecholaminergic polymorphic ventricular tachycardia in tactical athletes requires personalized strategies, considering their genetic profile, treatment response, and the critical responsibilities of their profession.
• In patients with early-onset arrhythmias, a low threshold for genetic testing may be considered to identify potential genetic cardiac conditions and guide management.

## Introduction

Catecholaminergic polymorphic ventricular tachycardia (CPVT) is a rare genetic disorder marked by adrenergically driven ventricular arrhythmias, typically presenting in childhood or early adulthood and often triggered by physical exertion or emotional stress, potentially leading to syncope or sudden death. CPVT arises from calcium handling disruptions in cardiac myocytes, crucial for stable cardiac excitation and contraction. Although *RYR2* gene variants are most commonly linked to CPVT, emerging evidence suggests that variants in the triadin (*TRDN*) gene, an essential protein in the cardiac sarcoplasmic reticulum, may cause similar arrhythmic syndromes.^1^^,^^2^ *TRDN* is a protein integral to the cardiac sarcoplasmic reticulum, and pathogenic biallelic variants have been implicated in a spectrum of often overlapping arrhythmogenic disorders, including CPVT, long QT syndrome, idiopathic ventricular tachycardia, and fibrillation.^3^^,^^4^ In these cases, biallelic disease–causing variants (ie, homozygous or compound heterozygous), result in the “knockout” of all protein expression and the phenotype tends to be severe.

We report a unique case of early-onset atrial fibrillation (AF) and mild CPVT due to a heterozygous truncating *TRDN* variant in a tactical athlete, specifically a firefighter whose profession requires high levels of physical fitness and the capacity to perform under unpredictable, high-stress conditions, often in life-threatening situations.^5^ This case suggests that heterozygous *TRDN* variations may contribute to a mild arrhythmia phenotype, raising considerations for management of potentially life-threatening arrhythmias due to poorly understood genotypes.

## Case report

**Figure 1:**
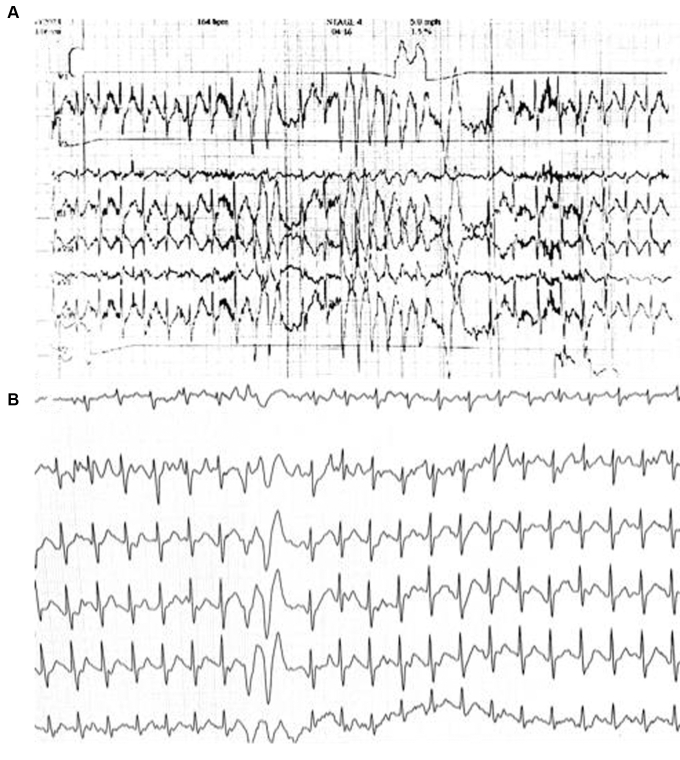
Baseline cardiopulmonary exercise test (CPET). The initial CPET showed suboptimal cardiac performance, multiple episodes of (**A**) nonsustained ventricular tachycardia and (**B**) couplets.

A 42-year-old male firefighter underwent a routine cardiopulmonary exercise test (CPET) sponsored by his local fire department. During exercise, the test demonstrated nonsustained polymorphic ventricular tachycardia, accompanied by palpitations and lightheadedness, leading to early termination. His submaximal oxygen consumption was measured at 41 mL/kg/min at the time (Figure 1). His medical history was significant for symptomatic recurrent paroxysmal AF at age 30 years, intolerant to flecainide, and he ultimately underwent catheter ablation with pulmonary vein isolation. He was clinically arrhythmia-free until 2 months before the CPET, when he presented to the hospital after having 2 episodes of highly symptomatic AF requiring electrical cardioversion.

**Figure 2:**
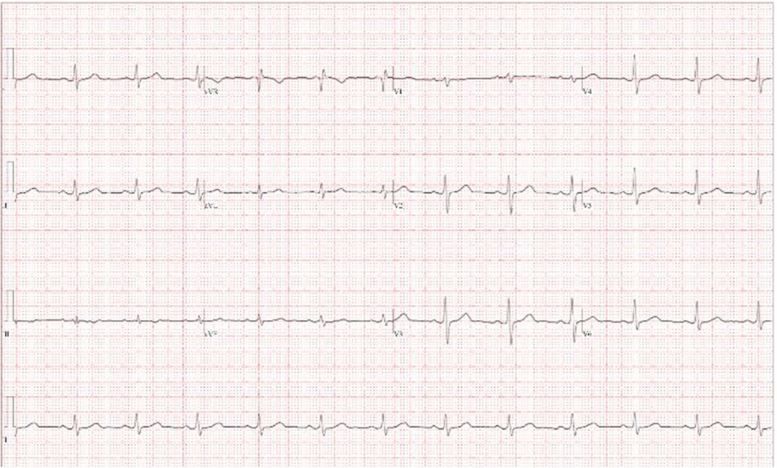
Baseline electrocardiogram.

After the CPET, he saw his local cardiologist. During the previous several months, he felt similar symptoms with intense exercise and during fire alarms, which he attributed to brief episodes of AF. Baseline physical and laboratory examination revealed no abnormal findings. Baseline electrocardiogram showed sinus rhythm without T-wave inversion and normal QT interval (Figure 2). An echocardiogram revealed normal left ventricular function with no structural abnormalities. A coronary angiogram revealed no evidence of coronary artery disease. He was advised to restrict exercise without a clear diagnosis and was subsequently referred to our electrophysiology team for further evaluation. There was no family history of sudden cardiac or unexplained death. In addition, there was no known history of early-onset arrhythmias; however, his parents died in their 40s due to a tragic accident. He also had no children or siblings. Cardiac magnetic resonance imaging indicated a structurally normal heart with no late gadolinium enhancement and a cardiac positron emission tomography scan revealed no signs of inflammation to exclude myocarditis.

Due to recurrent highly symptomatic AF, he was restarted on flecainide and nadolol for suspicion of CPVT while genetic testing was pending, and he subsequently underwent repeat AF ablation. During the procedure, all previously isolated veins were found to be reconnected with normal voltage. All veins were re-isolated and cavotricuspid isthmus ablation was also performed due to an inducible typical atrial flutter. No ventricular arrhythmias were induced during a ventricular stimulation protocol with and without isoproterenol.

Meanwhile, subsequent genetic testing via the GeneDx arrhythmia and cardiomyopathy panels (GeneDX LLC, Stamford, CT) identified a heterozygous variation in the *TRDN* gene [c.368_370del, p.(D123del)]—a variant of unknown significance. After these results, nadolol was continued and flecainide was discontinued per the patient’s desire, due to mild fatigue.

Through shared decision making, an implantable loop recorder was inserted for long-term arrhythmia monitoring. A repeat CPET with burst protocol after 3 months on nadolol showed no ventricular ectopy, with an oxygen consumption maximum of 35 mL/kg/min, despite no exercise training. He was then continued on nadolol with no exercise or occupational restrictions. At 2-year follow-up, the patient remains asymptomatic with no arrhythmia on long-term monitoring.

## Discussion

This case illustrates a mild CPVT associated with a heterozygous variation in *TRDN* in a middle-aged firefighter, manifesting as brief polymorphic ventricular tachycardia and early-onset AF. *TRDN* encodes a key protein in cardiac and skeletal muscle excitation-contraction coupling, essential for calcium handling.^6^ First reported in 2012, *TRDN* variation have been linked to a severe arrhythmic phenotype termed “triadin knockout syndrome,” marked by polymorphic ventricular tachycardia, T-wave inversions, and prolonged QT interval.^7^ This syndrome primarily involves biallelic variations causing total protein loss, resulting in aggressive arrhythmias in infancy or adolescence. Our case, however, suggested that even heterozygous *TRDN* variation, as in [c.368_370del, p.(D123del)], may present a mild CPVT phenotype. This implies that a single functional copy of TRDN may not fully sustain normal calcium homeostasis.

The Expert Consensus Statement on the State of Genetic Testing for Cardiac Diseases, endorsed by the European Heart Rhythm Association, Heart Rhythm Society, Asia Pacific Heart Rhythm Society, and Latin American Heart Rhythm Society, recognized *TRDN* as a gene associated with CPVT, noting its autosomal recessive inheritance pattern and its role in calcium release unit remodeling. In 2020, Sarquella-Brugada and colleagues^8^ identified 14 genetic variants in *TRDN* that have been reported to date. Most cases occurred in infants and adolescents; variants were either homozygous or compound heterozygous.

To the best of our knowledge, this is the first case report of a patient with clinical symptoms potentially linked to a heterozygous *TRDN* variant [c.368_370del, p.(D123del)], raising the possibility that a single functional copy of *TRDN* may not fully sustain normal cardiac calcium homeostasis. Similar findings have been observed in heterozygous forms of calsequestrin (*CASQ2)* variants that may have milder forms of CPVT.^9^ However, the evidence remains insufficient to definitively attribute the patient’s clinical manifestations to this variant, as it remains classified as a variant of uncertain significance. Additional reports of individuals carrying the same heterozygous variation with similar phenotypes would be necessary to establish clinical significance. Although the clinical presentation in this case is compelling, the potential contribution of other untested genetic variations cannot be excluded. Without living relatives for co-segregation studies, induced pluripotent stem cells could provide insight into the variation’s molecular mechanisms, although resources for this approach were not available in this case.

AF may be the first manifestation of an inherited cardiac condition in young adults, such as Brugada syndrome or long QT syndrome, although overall yield of routine genetic testing may be low.^10^^,^^11^ To date, *TRDN* variants have been linked to long QT syndrome but not AF.^12^ Calcium dysregulation in myocytes could potentially predispose individuals to atrial arrhythmias, suggesting a need for further research to explore this link and assess other monogenic and polygenic contributors to AF, especially in younger patients without traditional risk factors.

For athletes with CPVT who were previously symptomatic, return to play may be considered after confirming appropriate therapy, as outlined in the 2024 Heart Rhythm Society Expert Consensus Statement on Arrhythmias in the Athlete.^10^ However, management varies between competitive and tactical athletes. Competitive athletes typically face predictable physical demands and can modulate their activities accordingly. In contrast, tactical athletes, such as firefighters or military personnel, must perform under unpredictable, high-stress conditions, where cardiac stability is crucial for their safety and that of others.^13^ In this case, our patient experienced palpitations during emergency calls, possibly due to nonsustained polymorphic ventricular tachycardia. With the ongoing use of nadalol,^14^^,^^15^ suppression of ventricular ectopy during repeat burst protocol CPET,^16^ and ongoing monitoring with an implantable loop recorder demonstrating no AF or ventricular arrhythmia recurrence, he eventually returned to full duty without exercise restrictions. He was advised to stay alert for any recurrence of symptoms and to ensure an emergency action plan was in place, including access to an automated external defibrillator.

## Conclusion

This is the first documented case of early-onset AF and a mild form of CPVT in a patient with heterozygous *TRDN* variant. However, given that this report involves only a single patient, the clinical significance of this variant remains uncertain. For tactical athletes, such cases emphasize the importance of thorough risk assessment and rhythm stability verification to support safe return-to-duty decisions. Further research is necessary to better understand the potential role of *TRDN* variants in arrhythmia predisposition and to guide management strategies for affected individuals.

## Disclosures

Dr. Roston is a consultant for Cardurion Pharma and a consultant and advisory board member for Solid Biosciences. Dr. Krahn is an advisory Board member for Tenaya Pharmaceuticals. Dr. Lakkireddy is a consultant for Abbott Vascular, Biotronik, BioSense Webster, Medtronic, Boston Scientific, Atricure, Acutus, and Northeast Scientific. Dr. Pothineni is a consultant for Medtronic and Biosense Webster; the rest of the authors have no conflicts of interest.
